# Clinical characteristics of extremely elderly versus normal elderly patients with chronic limb-threatening ischemia: a single-center real-event retrospective study

**DOI:** 10.1186/s40001-025-03532-0

**Published:** 2025-12-20

**Authors:** Ke Wang, Haijun Wei, Qiqi Wang, Wei Huang, Yang Liu, Chunshui He

**Affiliations:** Department of Vascular Surgery, Hospital of Chengdu University of Traditional Chinese Medicine, Chengdu, 610075 Sichuan China

**Keywords:** Peripheral vascular disease, Extreme old age, Adverse limb events, Complications, Quality of life, Propensity score matching

## Abstract

**Background:**

The benefit of endovascular therapy (EVT) for chronic limb-threatening ischemia (CLTI) in patients aged ≥ 80 years (extremely elderly) is debated. This study compared outcomes between extremely elderly and younger elderly (60–79 years) patients.

**Methods:**

In this retrospective study, 367 patients (133 ≥ 80y, 234 60-79y) undergoing EVT were analyzed. Propensity score matching balanced baseline characteristics. Primary endpoints were one-year major adverse limb events (MALEs) and mortality. Univariate Cox analysis identified MALE-associated factors.

**Results:**

After matching, the ≥ 80y group had more complex lesions (longer occlusions, poorer runoff). Their one-year freedom from MALEs (44.721% vs 61.697%, p = 0.053) and freedom from mortality rates(77.191% vs 85.628%, p = 0.215) were lower. Univariate analysis indicated age ≥ 80y (HR = 1.55, 95% CI 1.01–2.38, P = 0.043) and TASC II C/D lesions (HR = 1.26, 95% CI 1.02–1.55, P = 0.035) increased MALE risk, while drug-coated balloons((HR = 0.69, 95% CI 0.56–0.85, P = 0.001).

and Lipid lowering agents usage (HR = 0.59, 95% CI 0.36–0.98, P = 0.041) were protective. Postoperative psychiatric symptoms were more frequent in the ≥ 80y group. Critically, both groups showed significant and comparable improvements in pain scores and functional capacity after EVT.

**Conclusions:**

Despite higher risks, EVT meaningfully improves quality of life in extremely elderly CLTI patients. Treatment should not be denied based on age alone, but should be individualized considering the higher anatomical complexity.

## Background

The incidence of peripheral vascular disease (PAD) is on the rise worldwide. Late-stage PAD is primarily characterized by chronic limb-threatening ischemia. When patients develop symptoms of limb-threatening ischemia, they may experience pain, reduced mobility, and chronic ulcers. In the later stages, amputation and death may occur [1]. The treatment of critical limb ischemia (CLTI) is primarily through open bypass or endovascular treatment to achieve blood flow reconstruction of the ischemic limb. Given that the majority of patients with PAD are elderly, open bypass is not a viable option for some frail elderly patients due to the high risk and trauma involved. Consequently, endovascular treatment is often the preferred approach for patients with PAD [2]. For the extremely elderly, in addition to the potential for restenosis of blood vessels in the late stage of endovascular treatment, the intraoperative complications (cardiovascular and cerebrovascular accidents, renal insufficiency, etc.) should be considered. For these reasons, extremely elderly patients present a significant challenge to surgeons in clinical practice.

The financial burden of endovascular therapy is significant for China’s aging population, which is growing at an alarming rate [3, 4]. In the context of reduced mobility and self-care in the elderly population, the value of such a costly and risky procedure in the extremely elderly patient becomes a crucial question. If the patient does not benefit from the surgery, the inappropriateness of this type of treatment is evident. However, there is a paucity of detailed analyses of the literature on this topic in China. In this study, we sought to explore the benefits of endovascular revascularization in extreme old age by including patients with chronic ischemia of the lower limb arteries in a retrospective analysis. We also compared and contrasted the general condition, surgical outcome, complications, and ability to live in normal old age (60–80 years old) and extreme old age (≥ 80 years old) patients.

## Patients and methods

### Patient population

All patients who were initially diagnosed with chronic limb-threatening ischemia (CLTI) and subsequently underwent endovascular therapy at the Affiliated Hospital of Chengdu University of Traditional Chinese Medicine between 2018 and 2023 were included in the study. Patients were diagnosed with lower extremity atherosclerotic occlusive disease if they presented with rest pain and/or ischemic tissue loss consistent with Rutherford stages 2 to 5.

Inclusion criteria: (1) The presence of severe stenosis (≥ 70%) or occlusive disease in the femoropopliteal artery; (2) Successful restoration of lumen patency (residual stenosis < 30% and no infinite-flow entrapment formation) by existing endovascular therapies, either in primary femoropopliteal artery lesions or in-stent restenosis, was sufficient for enrollment. Additionally, those who had previously undergone treatment but experienced failure due to the inability of the guidewire to pass through the lesion were eligible for enrollment if they had undergone a subsequent endovascular intervention with successful results. The following individuals were excluded from the study: (1) Patients with acute arterial thrombosis; (2) Affected limbs previously treated with femoropopliteal bypass surgery; (3) Patients with serious lesions such as liver failure; (4) Patients with chronic ischemia of the ipsilateral limb prior to the study period as well as patients requiring immediate amputation due to massive tissue loss or infection were excluded. The study was approved by the Ethics Committee of the Affiliated Hospital of Chengdu University of Traditional Chinese Medicine. Patients were informed about the use of their pseudonymous medical data. No patients declined to have their data utilized in this study.

### Variables evaluated

**Fig. 1:**
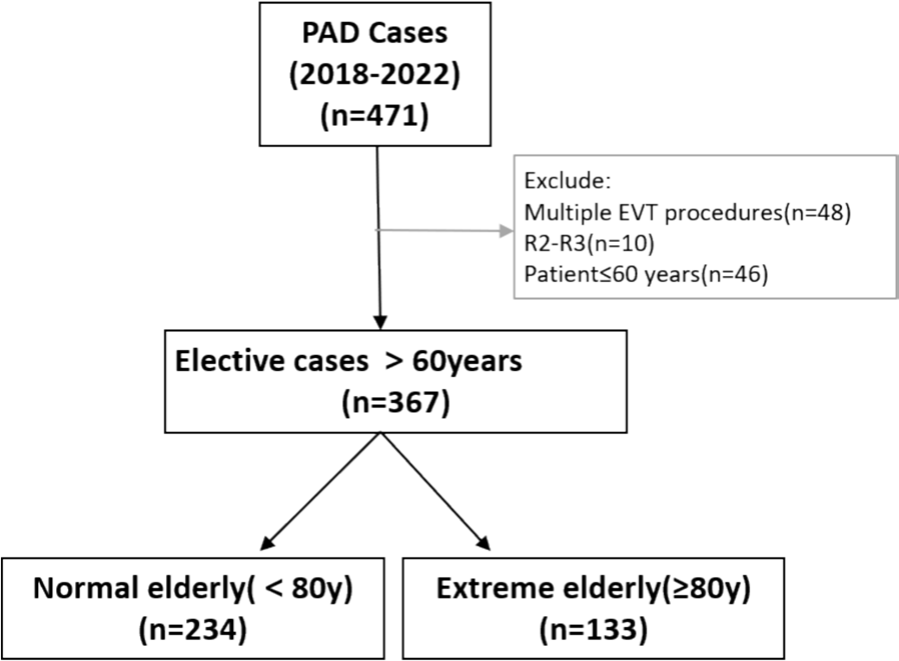
Patient selection process. *PAD* peripheral vascular disease *EVT* endovascular therapy

The patients were retrospectively evaluated with regard to their basic condition, lesion characteristics, surgical procedure, and postoperative oral medications. The items of basic condition included age, gender, underlying disease, and basic information such as ABI, Rutherford classification, history of previous endovascular treatment, previous ipsilateral debridement, and presence of contralateral lesion regarding CLTI. The characterization of the lesion was based on the patient’s preoperative images (CTA or DSA), which included the location and nature of the lesion (stenosis or occlusion), lesion length, TASC classification, calcification, and infrapopliteal outflow tract. The infrapopliteal artery was considered to be patent with less than 30% stenosis throughout the entire length of the infrapopliteal outflow tract (Fig. 1). The procedure describes the manner of passage through the lesion, including whether a drug-coated balloon was used, whether a stent was placed, whether volume reduction was employed, and the number of infrapopliteal outflow tracts after the procedure.

### Study end points

All patients were followed up for more than one year, with results registered at four intervals: 30 days, 3 months, 6 months, and 1 year. The primary endpoint of this study was a pre-specified composite outcome, defined as the occurrence of Major Adverse Limb Events (MALEs) within 12 months post-procedure. MALEs included major amputation (amputation above the ankle level) and clinically-driven target vessel revascularization (such as surgical or endovascular re-intervention). All endpoint criteria were explicitly defined in the study protocol. Furthermore, the Barthel Index was used to assess patients’ activities of daily living. Assessments were conducted by uniformly trained research nurses at baseline (upon hospital admission), at discharge, and during telephone follow-ups at 6 and 12 months postoperatively, using a standardized Chinese version of the Barthel Index scale, which has been validated for good reliability and validity in our study population. The Visual Analog Scale (VAS) was employed to evaluate rest pain in the affected limb. Patients were instructed by the same research nurse to mark their pain level on a 10-cm line, with ‘0’ indicating “no pain” and ‘10’ indicating “worst pain imaginable”; the corresponding numerical value (in centimeters) was recorded. VAS assessments were performed one day before surgery and within one day after the procedure.

### Statistical analysis

Analyses were performed using the SPSS software (versions 20.0 and 23.0; SPSS). Survival graphs with 95% CIs were created in Stata Statistical Software Release 15 (StataCorp). Continuous data are given as mean (SD) or, if there is no normal distribution, as median with interquartile range; categorical data are presented as counts (percentages). The χ2 test or, in cases of cell size less than 5, the Fisher exact test, were used to compare the variables. Kaplan–Meier analysis was used to evaluate survival, and the survival curves were compared using the log-rank test. Statistical significance was defined as P < 0.05.

Propensity score matching (PSM) at a 1:1 ratio was applied to reduce confounding between the extremely elderly and normal elderly groups, using a caliper width of 0.1 standard deviations. Matching variables included male, diabetes, atrial fibrillation and current smoker assessed by the Bollinger score. This approach was used to balance baseline characteristics between groups and minimize selection bias. In addition to PSM, Univariable Cox proportional hazards models were conducted within the matched cohort to adjust for any residual imbalances and further evaluate the independent association between age and clinical outcomes.

## Results

### Baseline characteristics

A total of 367 patients were included in this study, of which 133 were aged 80 years or older and 234 were aged less than 80 years. As illustrated in Table 1, the mean age of patients in the extreme elderly group was 84.98 ± 3.61 years, in contrast to 70.78 ± 6.52 years in the normal elderly group. The proportion of males in the extreme elderly patients was significantly lower than that in the normal elderly group (74.5% vs. 57.14%, P = 0.001). The rates of hypertension, diabetes mellitus, hyperlipidemia, chronic renal failure, and cerebrovascular diseases were lower in the extreme elderly group than in the normal elderly group. A statistically significant difference was observed in diabetes mellitus between the two groups (37.59% vs. 61.97%, P < 0.001). The prevalence of atrial fibrillation (15.79%) was significantly higher in the extreme elderly group than in the normal elderly group (6.84%), with a statistically significant difference (P = 0.006). Additionally, patients in the normal elderly group were more likely to smoke compared to the extreme elderly group (18.05% vs. 32.05%, P = 0.018). The prevalence of heart failure and ischemic heart disease was higher in the extreme elderly group than in the normal elderly group, and the ejection fraction (EF%) was lower in the extreme elderly group than in the normal elderly group. Propensity score matching was used to achieve balanced baseline characteristics across both groups. No statistically significant differences were observed between the two groups in terms of bilateral symptoms, history of previous revascularization, previous minor amputations, and Rutherford grading.

**Table 1:** Baseline

Variable	Before PSM					After PSM				
	Total (n = 367)	< 80 (n = 234)	≥ 80 (n = 133)	Statistic	*P*	Total (n = 240)	Age < 80 (n = 120)	Age ≥ 80 (n = 120)	Statistic	*P*
Age, years	75.92 ± 8.86	70.78 ± 6.52	84.98 ± 3.61	− 26.85	** <.001**	78.25 ± 8.68	71.32 ± 6.44	85.17 ± 3.60	− 20.57	** <.001**
Male, n (%)	248 (67.57)	172 (73.50)	76 (57.14)	10.36	**0.001**	146 (60.83)	74 (61.67)	72 (60.00)	0.07	0.791
BMI(kg/m^2^)	22.59 ± 8.45	23.05 ± 10.26	21.78 ± 3.35	1.39	0.166	22.39 ± 10.25	23.03 ± 14.08	21.76 ± 3.45	0.96	0.339
Diabetes, n (%)	195 (53.13)	145 (61.97)	50 (37.59)	20.23	** <.001**	68 (28.33)	31 (25.83)	37 (30.83)	0.74	0.390
Atrial fibrillation, n (%)	37 (10.08)	16 (6.84)	21 (15.79)	7.50	**0.006**	29 (12.08)	16 (13.33)	13 (10.83)	0.35	0.552
Hypertension, n (%)	264 (71.93)	173 (73.93)	91 (68.42)	1.28	0.259	165 (68.75)	85 (70.83)	80 (66.67)	0.48	0.486
Hyperlipidaemia, n (%)	164 (44.69)	108 (46.15)	56 (42.11)	0.56	0.453	104 (43.33)	54 (45.00)	50 (41.67)	0.27	0.602
Current smoker, n (%)	99 (26.98)	75 (32.05)	24 (18.05)	8.45	**0.004**	80 (33.33)	39 (32.50)	41 (34.17)	0.08	0.784
End-stage renal disease, n (%)	41 (11.17)	27 (11.54)	14 (10.53)	0.09	0.767	22 (9.17)	9 (7.50)	13 (10.83)	0.80	0.371
Cerebrovascular disease, n (%)	97 (26.43)	65 (27.78)	32 (24.06)	0.60	0.438	46 (19.17)	19 (15.83)	27 (22.50)	1.72	0.190
Congestive heart failure, n (%)	52 (14.17)	30 (12.82)	22 (16.54)	0.97	0.326	33 (13.75)	13 (10.83)	20 (16.67)	1.72	0.189
Ischaemic heart disease, n (%)	106 (28.88)	65 (27.78)	41 (30.83)	0.38	0.536	59 (24.58)	23 (19.17)	36 (30.00)	3.80	0.051
EF%	65.05 ± 8.06	65.38 ± 8.36	64.87 ± 7.97	−0.27	0.786	64.49 ± 8.54	63.57 ± 8.78	65.38 ± 8.36	−0.80	0.429
Bilateral PAD, n (%)	216 (58.86)	135 (57.69)	81 (60.90)	0.36	0.548	142 (59.17)	69 (57.50)	73 (60.83)	0.28	0.599
Previous peripheral Revascularisation of target limb, n (%)	103 (28.07)	61 (26.07)	42 (31.58)	1.28	0.259	63 (26.25)	25 (20.83)	38 (31.67)	3.64	0.056
Previous minor target limb amputation, n (%)	16 (4.36)	13 (5.56)	3 (2.26)	2.21	0.137	10 (4.17)	7 (5.83)	3 (2.50)	1.67	0.196
Rutheford Category, n (%)				6.35	0.174				6.79	0.147
2	10 (2.72)	8 (3.42)	2 (1.50)			7 (2.92)	5 (4.17)	2 (1.67)		
3	70 (19.07)	51 (21.79)	19 (14.29)			47 (19.58)	30 (25.00)	17 (14.17)		
4	114 (31.06)	67 (28.63)	47 (35.34)			77 (32.08)	34 (28.33)	43 (35.83)		
5	155 (42.23)	99 (42.31)	56 (42.11)			96 (40.00)	46 (38.33)	50 (41.67)		
6	18 (4.90)	9 (3.85)	9 (6.77)			13 (5.42)	5 (4.17)	8 (6.67)		
Ankle-brachial index	0.43 ± 0.28	0.47 ± 0.30	0.36 ± 0.24	1.65	0.103	0.39 ± 0.25	0.40 ± 0.26	0.37 ± 0.24	t = 0.46	0.650

**Table 2:** Lesion characteristics

Variable	Before PSM					After PSM				
	Total (n = 367)	< 80 (n = 234)	≥ 80 (n = 133)	Statistic	*P*	Total (n = 240)	Age < 80 (n = 120)	Age ≥ 80 (n = 120)	Statistic	*P*
Lesion				0.33	0.566				1.96	0.161
De novo, n (%)	330 (89.92)	212 (90.60)	118 (88.72)			220 (91.67)	113 (94.17)	107 (89.17)		
Restenotic, n (%)	37 (10.08)	22 (9.4)	15 (11.28)			20 (8.33)	7 (5.83)	13 (10.83)		
Target lesion location										
Superficial femoral artery, n (%)	347 (94.55)	219 (93.59)	128 (96.24)	1.16	0.282	221 (92.08)	106 (88.33)	115 (95.83)	4.63	**0.031**
Popliteal artery, n (%)	177 (48.23)	102 (43.59)	75 (56.39)	5.57	**0.018**	119 (49.58)	52 (43.33)	67 (55.83)	3.75	0.053
Occluded, n (%)	292 (79.56)	185 (79.06)	107 (80.45)	0.10	0.751	193 (80.42)	97 (80.83)	96 (80.00)	0.03	0.871
Total occluded lesion length, mm	140.89 ± 127.30	130.26 ± 120.75	159.59 ± 136.53	− 2.06	**0.040**	142.65 ± 126.45	126.21 ± 112.04	159.08 ± 137.90	− 2.03	**0.044**
TASC II, n (%)				5.01	0.171				3.54	0.316
A	39 (10.63)	26 (11.11)	13 (9.77)			24 (10.00)	11 (9.17)	13 (10.83)		
B	60 (16.35)	44 (18.80)	16 (12.03)			38 (15.83)	24 (20.00)	14 (11.67)		
C	170 (46.32)	109 (46.58)	61 (45.86)			111 (46.25)	55 (45.83)	56 (46.67)		
D	98 (26.70)	55 (23.50)	43 (32.33)			67 (27.92)	30 (25.00)	37 (30.83)		
Calcification, n (%)				10.60	**0.014**				10.39	0.016
0	58 (15.80)	42 (17.95)	16 (12.03)			34 (14.17)	20 (16.67)	14 (11.67)		
1	101 (27.52)	73 (31.20)	28 (21.05)			67 (27.92)	41 (34.17)	26 (21.67)		
2	111 (30.25)	68 (29.06)	43 (32.33)			78 (32.50)	38 (31.67)	40 (33.33)		
3	97 (26.43)	51 (21.79)	46 (34.59)			61 (25.42)	21 (17.50)	40 (33.33)		
The number of patent tibial arteries(pre-operation), n (%)				14.62	**0.002**				14.61	**0.002**
0	173 (47.14)	98 (41.88)	75 (56.39)			117 (48.75)	48 (40.00)	69 (57.50)		
1	85 (23.16)	53 (22.65)	32 (24.06)			52 (21.67)	25 (20.83)	27 (22.50)		
2	60 (16.35)	41 (17.52)	19 (14.29)			39 (16.25)	22 (18.33)	17 (14.17)		
3	49 (13.35)	42 (17.95)	7 (5.26)			32 (13.33)	25 (20.83)	7 (5.83)		

### Lesion characteristics, surgical procedure, postoperative complications and postoperative medication

As demonstrated in (Table 2), after PSM, a comparison of lesion locations revealed that, in addition to a higher prevalence of lesions in the superficial femoral artery, patients in the extreme old age group exhibited a significantly higher incidence of lesions in the superficial femoral artery compared to the normal old age group (95.83% vs. 88.33%, P = 0.031). Although the rate of developing occlusive lesions was not significantly different between the two groups, the total occlusion length was significantly higher in the extreme elderly group than in the normal elderly group (126.21 ± 112.04 vs 159.08 ± 137.90,P = 0.044). The condition of the infrapopliteal outflow tract had a greater impact on the patency of the femoropopliteal artery. To this end, we also compared the infrapopliteal outflow tracts of the patients in each group. This revealed that the patients in the extreme old age group had worse infrapopliteal outflow tracts than those in the normal old age group.

Intraoperatively, no significant differences were observed between Lesion crossed with guidewire, stenting, drug-coated balloon use, volume reduction, thrombus, and thrombus management in either group (Table 3). Despite surgical intervention, patients in the extreme geriatric group exhibited poorer below-knee outflow tracts than those in the normal intervention group. Intraoperatively, distal embolization occurred in five cases without vessel rupture in the extreme elderly group, and distal embolization occurred in six cases and vessel rupture in one case in the normal elderly group. The postoperative complication statistics (Table 4) indicate that puncture point hemorrhage and hematoma were less frequent in the extreme elderly group than in the normal elderly group. The incidence of pseudoaneurysms at the puncture point was higher in the extreme geriatric group. The difference in the incidence of abnormalities in liver function, abnormalities in renal function, cerebrovascular accidents, and acute heart failure between the two groups was minimal. However, the proportion of psychiatric symptoms in the extreme geriatric group was significantly higher than that in the normal geriatric group (15.83% vs. 3.33%, P = 0.001).Table 5 presents the medications taken by the two groups. The use of lipid-lowering drugs (38.98% vs. 26.67%, P = 0.043) differed significantly between the two groups (after PSM), while no statistically significant differences were observed for the remaining items.

**Table 3:** Intraoperative characteristics

Variable	Before PSM					After PSM				
	Total (n = 367)	< 80 (n = 234)	≥ 80 (n = 133)	Statistic	*P*	Total (n = 240)	Age < 80 (n = 120)	Age ≥ 80 (n = 120)	Statistic	*P*
Lesion crossed with guidewire				8.804	**0.012**				–	0.622
True lumen, n (%)	266 (72.48)	178 (76.07)	88 (66.17)			175 (72.92)	92 (76.67)	83 (69.17)		
Subintimal, n (%)	18 (4.90)	6 (2.56)	12 (9.02)			6 (2.5)	2 (0.167)	4 (3.33)		
Bidirectional subintimal, n (%)	83 (22.62)	50 (21.37)	33 (24.81)			59 (24.58)	26 (21.67)	33 (27.50)		
DCB, n (%)	245 (66.76)	159 (67.95)	86 (64.66)	0.41	0.521	151 (62.92)	72 (60.00)	79 (65.83)	0.88	0.350
Coverage of lesion patterns										
All, n (%)	175 (47.68)	116 (49.57)	59 (44.36)	0.92	0.337	112 (46.67)	58 (48.33)	54 (45.00)	0.27	0.605
Part, n (%)	69 (18.80)	42 (17.95)	27 (20.30)	0.31	0.579	39 (16.25)	14 (11.67)	25 (20.83)	3.70	0.054
Stent, n (%)				–	0.236				–	0.745
Covered stent	1 (0.27)	1 (0.43)	0 (0.00)			1 (0.42)	1 (0.83)	0 (0.00)		
Drug coated stent	2 (0.54)	2 (0.85)	0 (0.00)			1 (0.42)	1 (0.83)	0 (0.00)		
Bare metal stent	70 (19.07)	43 (18.38)	27 (20.30)			47 (19.58)	22 (18.33)	25 (20.83)		
Presence of thrombus, n(%)	32 (8.72)	19 (8.12)	13 (9.77)	0.29	0.589	30 (12.50)	19 (15.83)	11 (9.17)	2.44	0.118
Whether to handle thrombosis, n(%)	27 (6.54)	14 (5.56)	13 (9.77)	1.79	0.18	22 (10.42)	12 (12.5)	10 (8.33)	0.063	0.803
Method, n(%)										
Angiojet, n(%)	22 (5.99)	10 (4.27)	12 (9.02)	3.39	0.065	5 (2.08)	4 (3.33)	1 (0.83)	-	0.372
Roterex, n(%)	5 (1.36)	4 (1.71)	1 (0.75)	0.09	0.770	20 (8.33)	10 (8.33)	10 (8.33)	0	1
Atherectomy, n(%)	10 (2.72)	6 (2.56)	4 (3.01)	0.00	1.000	8 (3.33)	4 (3.33)	4 (3.33)	0.00	1.000
Distal embolization, n(%)	11 (3.00)	6 (2.56)	5 (3.76)	0.11	0.744	9 (3.75)	5 (4.17)	4 (3.33)	0.00	1.000
Perforation, n(%)	1 (0.27)	1 (0.43)	0 (0.00)	-	1.000	1 (0.42)	1 (0.83)	0 (0.00)	-	1.000
Dissection, n(%)	49 (13.35)	30 (12.82)	19 (14.29)	0.16	0.692	29 (12.08)	11 (9.17)	18 (15.00)	1.92	0.166
The number of patent tibial arteries (post-operation), n(%)				10.60	**0.014**				-	0.018
0	15 (4.09)	9 (3.85)	6 (4.51)			9 (3.75)	4 (3.33)	5 (4.17)		
1	168 (45.78)	98 (41.88)	70 (52.63)			111 (46.25)	47 (39.17)	64 (53.33)		
2	124 (33.79)	78 (33.33)	46 (34.59)			84 (35.00)	43 (35.83)	41 (34.17)		
3	60 (16.35)	49 (20.94)	11 (8.27)			36 (15.00)	26 (21.67)	10 (8.33)

**Table 4:** Post-operative complications

Variable	Before PSM					After PSM				
	Total (n = 367)	< 80 (n = 234)	≥ 80 (n = 133)	Statistic	*P*	Total (n = 240)	Age < 80 (n = 120)	Age ≥ 80 (n = 120)	Statistic	*P*
Bleeding and puncture point hematoma, n (%)	39 (10.63)	26 (11.11)	13 (9.77)	0.16	0.690	22 (9.17)	11 (9.17)	11 (9.17)	0.00	1.000
Pseudoaneurysm, n (%)	20 (5.45)	11 (4.70)	9 (6.77)	0.70	0.402	10 (4.17)	3 (2.50)	7 (5.83)	1.67	0.196
Cerebrovascular Accident, n (%)	25 (6.81)	16 (6.84)	9 (6.77)	0.00	0.979	11 (4.58)	3 (2.50)	8 (6.67)	2.38	0.123
Hepatic function abnormalities, n (%)	9 (2.45)	6 (2.56)	3 (2.26)	0.00	1.000	8 (3.33)	5 (4.17)	3 (2.50)	0.13	0.719
Abnormality of kidney function, n (%)	27 (7.36)	15 (6.41)	12 (9.02)	0.85	0.357	16 (6.67)	6 (5.00)	10 (8.33)	1.07	0.301
Acute heart failure, n (%)	30 (8.17)	19 (8.12)	11 (8.27)	0.00	0.960	18 (7.50)	8 (6.67)	10 (8.33)	0.24	0.624
Psychiatric symptom, n (%)	35 (9.54)	14 (5.98)	21 (15.79)	9.45	**0.002**	23 (9.58)	4 (3.33)	19 (15.83)	10.82	**0.001**

**Table 5:** Medication

Variable	Before PSM					After PSM				
	Total (n = 367)	< 80 (n = 234)	≥ 80 (n = 133)	Statistic	*P*	Total (n = 240)	Age < 80 (n = 120)	Age ≥ 80 (n = 120)	Statistic	*P*
Lipid lowering agents, n(%)	39 (10.63)	26 (11.11)	13 (9.77)	0.16	0.690	78 (32.77)	46 (38.98)	32 (26.67)	4.10	**0.043**
ACE inhibitor/ARB, n(%)	20 (5.45)	11 (4.70)	9 (6.77)	0.70	0.402	51 (21.25)	26 (21.67)	25 (20.83)	0.02	0.875
Beta-blocker, n(%)	25 (6.81)	16 (6.84)	9 (6.77)	0.00	0.979	36 (15.00)	19 (15.83)	17 (14.17)	0.13	0.718
Antiplatelet therapy, n(%)	9 (2.45)	6 (2.56)	3 (2.26)	0.00	1.000	197 (82.08)	102 (85.00)	95 (79.17)	1.39	0.239
DOAC, n(%)	27 (7.36)	15 (6.41)	12 (9.02)	0.85	0.357	31 (12.92)	17 (14.17)	14 (11.67)	0.33	0.564
Oral antidiabetics, n(%)	30 (8.17)	19 (8.12)	11 (8.27)	0.00	0.960	46 (19.17)	23 (19.17)	23 (19.17)	0.00	1.000
Sagrelate Hydrochloride Tablets, n(%)	35 (9.54)	14 (5.98)	21 (15.79)	9.45	**0.002**	190 (79.17)	97 (80.83)	93 (77.50)	0.40	0.525

### Cox proportional hazards analyses in matched cohorts

**Fig. 2:**
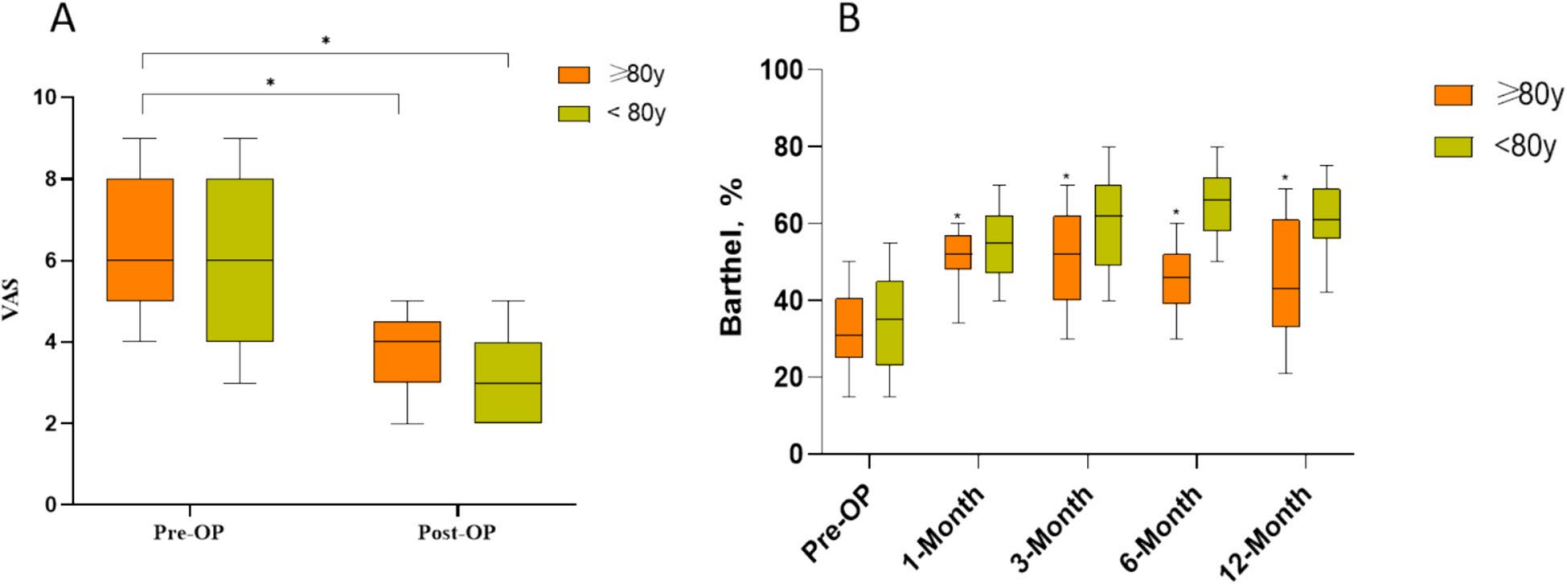
Post-operative pain and quality of life distribution compared with baseline (**A**) VAS score; **B** Barthel score (^*^P < 0.05)

**Fig. 3:**
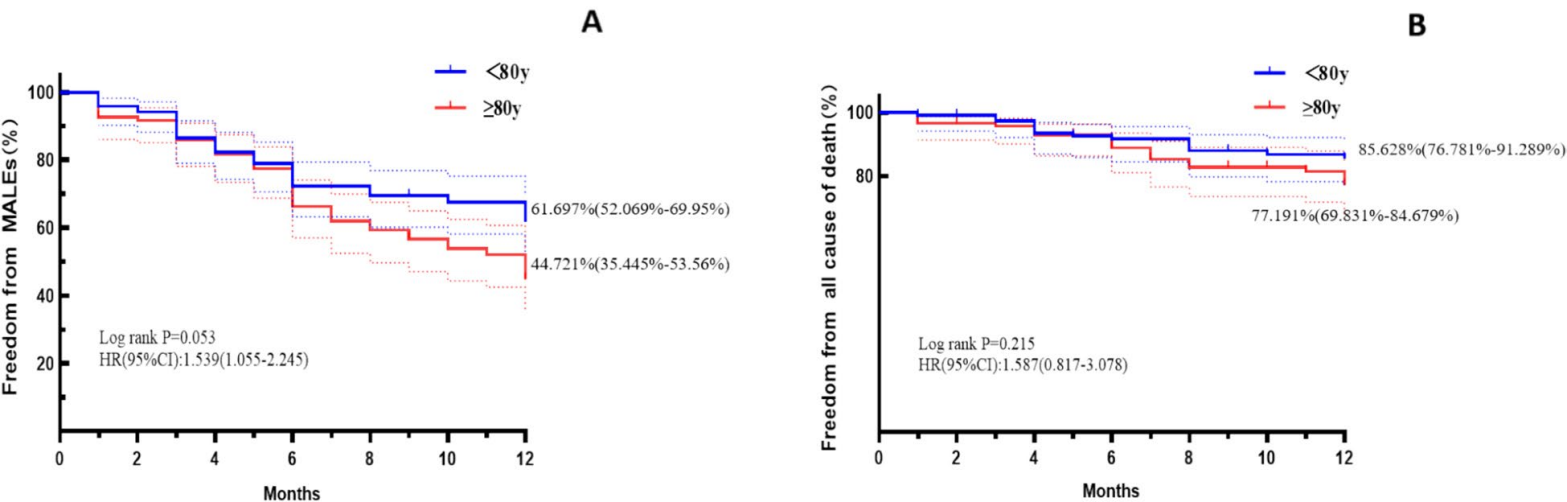
Kaplan–Meier analyses for 1-year outcomes: Major adverse limb events (A) and all cause of death (B)

**Fig. 4:**
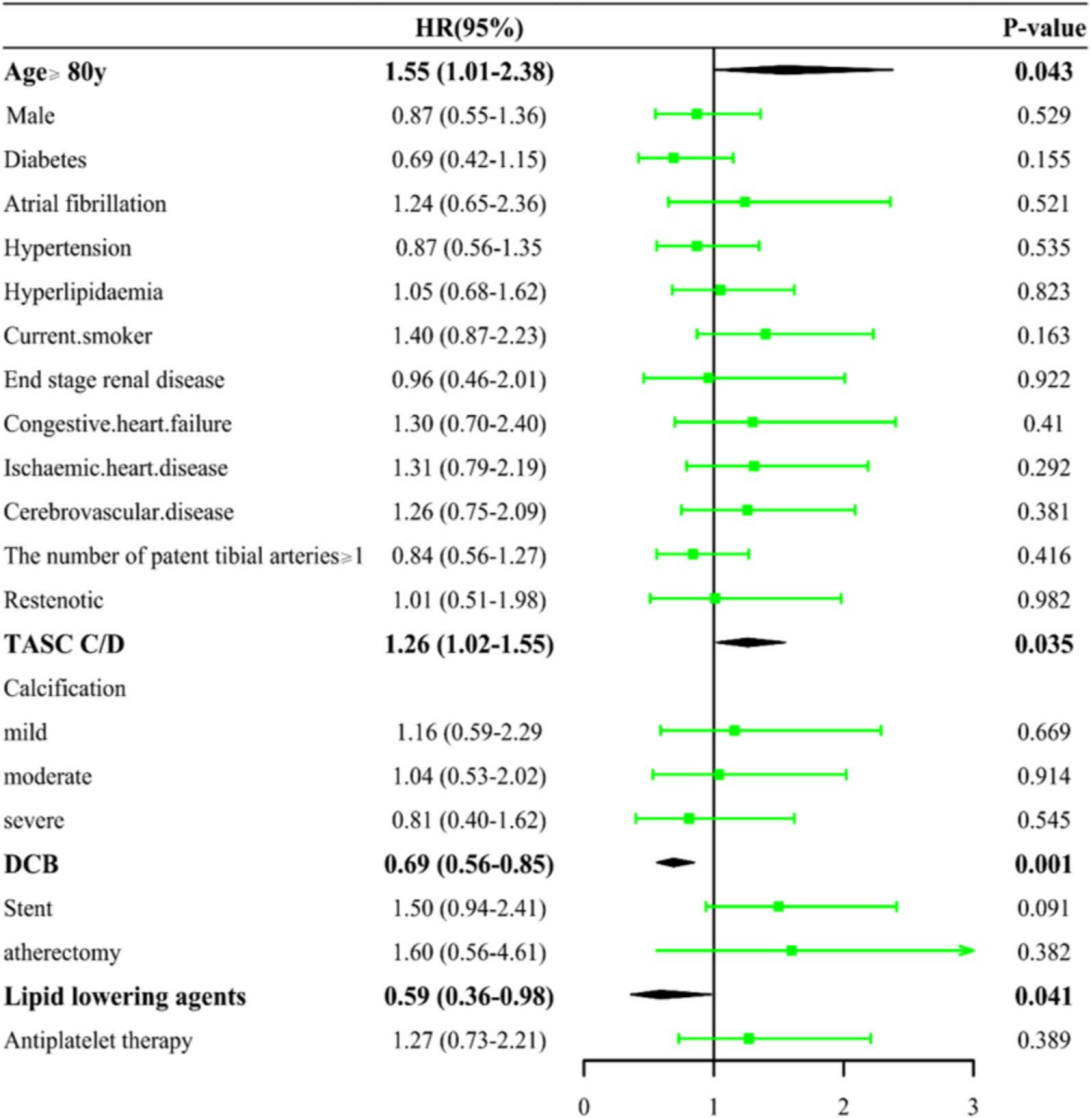
Forest plot of univariable Cox regression analysis for factors associated with major adverse limb events (MALEs)

According to the VAS scores recorded on the pre-operative day and post-operative day 1, endovascular therapy can alleviate the symptoms in both groups of PAD patients (Figure 2A). From the various follow-up time points after surgery, it can be observed that patients’ mobility and activity levels improved after discharge (Figure 2B). A 12-month follow-up of the patients in both groups revealed that the freedom from all-cause death rate was lower in the extreme elderly group than in the normal elderly group (77.191% vs 85.628%, p = 0.215, Fig. 3B) (Fig. 3). Additionally, the rate of freedom from major adverse limb events (MALEs) was lower in the extreme elderly group than in the normal elderly group (44.721% vs 61.697%, p = 0.053, Fig. 3A). As shown in Fig. 4, univariate Cox regression analysis of the matched cohort revealed that several variables were significantly associated with an increased risk of MALEs. Among them, age ≥ 80 years (HR = 1.55, 95% CI 1.01–2.38, P = 0.043) and TASC II type C/D lesions (HR = 1.26, 95% CI 1.02–1.55, P = 0.035) were identified as potential predictors. This means that, without adjusting for other factors, the risk of MALEs in extreme elderly patients (≥ 80 years) is 1.55 times higher than that in non-diabetic patients; while the risk for patients with TASC II type C/D femoropopliteal artery lesions is 1.26 times higher than that for patients with type A/B lesions. Stent implantation showed borderline statistical significance (HR = 1.50, 95% CI 0.94–2.41, P = 0.091). Other variables, such as gender and hypertension, showed no statistically significant associations in the univariate analysis of this study. Furthermore, drug-coated balloon (DCB) (HR = 0.69, 95% CI 0.56–0.85, P = 0.001) and lipid-lowering medications (HR = 0.59, 95% CI 0.36–0.98, P = 0.041) were associated with a reduced risk of MALEs.

## Discussion

In China, the prevalence of PAD is approximately 15% to 20% in people aged 70 years or older, and increases to approximately 50% in people aged 85 years or older [5]. With the aging of society, the prevalence of PAD in the population is expected to continue to increase, and this increase in prevalence places greater demands on the prevention and treatment of atherosclerotic occlusive disease of the lower extremities in the elderly, posing an unprecedented challenge to the healthcare system [5]. In the elderly population, extremely elderly patients have lower physical function than normal patients, resulting in poorer tolerance for surgery, which poses a great challenge to the surgeon. In the elderly population, extremely elderly patients have lower physical functioning than normal patients, resulting in poorer tolerance for surgery, which is a great challenge for the surgeon. This paper highlights the clinical characteristics of the extreme elderly population with PAD and the prognosis after endovascular therapy by comparing extreme elderly patients as well as patients in the normal elderly group, thus providing evidence on top of evidence-based medicine.

It is well established that underlying diseases such as hypertension, diabetes mellitus, and hyperlipidemia are causative factors for peripheral artery disease (PAD) [6, 7]. Consequently, the prevalence of these underlying diseases in patients with PAD tends to be significantly higher than in the general population. In addition, the older the patient, the higher the probability of having an underlying disease. However, the present study found that the rate of underlying diseases was significantly lower in extremely elderly PAD patients than in the normal elderly group, a result that is clearly inconsistent with the above common sense. Lida et al. found that underlying diseases such as hypertension and hyperglycemia were more prevalent in younger PAD patients [8]. Further, a study of Americans found that the prevalence of underlying diseases was significantly lower in extremely elderly carotid patients than in the normal elderly group. The prevalence of underlying diseases such as hypertension was significantly lower in the extremely elderly carotid patients than in the normal elderly group [9]. Disease prevalence was significantly less likely than in the normal older age group. These studies are consistent with the results of our data and do not refute the idea that underlying diseases such as hypertension are independent risk factors for PAD. Rather, they suggest that a high prevalence of underlying diseases undoubtedly contributes to an earlier age of onset of PAD.

The most crucial prognostic indicator following endovascular therapy is the rate of adverse limb events. In a study by Tanner I. Kim, it was found that the patency rate of endovascular therapy was 60% and the probability of freedom from major amputation was approximately 23% in extremely old patients (> 85 years). A significant reduction was also observed in extremely old patients in a study by B. In the present study, the rate of late adverse limb events was higher in the extremely old group than in the normal old group. In the present study, the extreme old age group exhibited a higher rate of late adverse limb events compared to the normal old age group. Previous studies have demonstrated that the degree of calcification of the lesion, the length of the lesion, and the poorer below-knee outflow tract play a significant role in inhibiting the patency rate after endovascular treatment of the femoropopliteal artery. The present study revealed that the lesion characteristics of patients in the extreme elderly group exhibited a significantly higher proportion of blocked lesion length and severe calcification than those in the normal elderly group. Additionally, the infrapopliteal outflow tract in the extreme elderly group was found to be inferior to that of the normal elderly group, both before and after the endovascular intervention. This aspect indicates that it is more difficult to open the vessel in extreme elderly patients with CLTI. Additionally, it provides insight into the higher rate of adverse limb events in extreme elderly patients.

The most crucial prognostic indicator following endovascular therapy is the rate of adverse limb events. In a study by Tanner I. Kim, it was found that the patency rate of endovascular therapy in extremely old patients (> 85 years) was 60% and the probability of freedom from major amputation was approximately 23% [10]. Similarly In a study by Tekin, N, it was found to be significantly lower in extremely old patients [11]. In the present study, the rate of adverse limb events in the later stages of life was higher in the extremely old group compared to the normal old group. Previous studies have demonstrated that the degree of calcification of the lesion, the length of the lesion, and the poorer below-knee outflow tract play a significant role in inhibiting the patency rate after endovascular treatment of the femoropopliteal artery. The present study revealed that patients in the extreme elderly group exhibited a significantly higher proportion of blocked lesion length and severe calcification than those in the normal elderly group. Additionally, the infrapopliteal outflow tracts in the extreme elderly group were found to be more severely impaired than those in the normal elderly group, both before and after endovascular intervention. This aspect indicates that it is more difficult to open the vessel in extreme elderly patients with peripheral artery disease (PAD). It also suggests that the higher rate of adverse limb events in extreme elderly patients can be attributed to this difficulty. As demonstrated in Yusuke Watanab’s study, an improved outflow tract in the infrapopliteal region can play a beneficial role in maintaining patency of the femoropopliteal artery [12]. This suggests that opening as many infrapopliteal vessels as possible may reduce the incidence of adverse limb events in extremely elderly patients, provided that the technique allow.

With regard to postoperative complications, this study revealed a marginal discrepancy in the incidence of puncture site hematoma, puncture site pseudoaneurysm, and hepatic and renal kinetic abnormalities between the two groups. This finding is consistent with previous studies. The literature indicates that the use of large amounts of contrast media may lead to the accumulation of contrast media in the brain, resulting in contrast encephalopathy. The imaging manifestations of this condition are suggestive of a large amount of contrast media accumulation in the patient’s brain [13, 14]. We observed that elderly patients presented with psychiatric abnormalities, such as lethargy and delirium, at a significantly higher rate compared to the normal elderly group. The symptoms gradually abated with enhanced postoperative hydration, suggesting that they may be contrast encephalopathy, a condition that has been less frequently reported in the literature.

In postoperative follow-up, all-cause mortality was also significantly higher in extremely elderly patients compared to the normal elderly group. These discussions collectively suggest that extreme elderly patients with PAD have poorer postoperative outcomes with endovascular therapy. Consequently, some scholars have proposed that since the hemodynamic outcomes of elderly patients with PAD treated conservatively are similar to those with endovascular therapy, revascularization may not be necessary in every case. This also supports the notion that extreme elderly patients with PAD have less to gain from endovascular therapy. Although extreme elderly patients have a reduced life expectancy and surgery may not be able to prolong their life expectancy, the reduction of pain symptoms and improvement in quality of life is certainly one of the aspects to be considered in the context of surgery. Previous studies have assessed the effectiveness of treatment by quality of life as assessed by VascuQoL-25 [15]. However, the limitations of this study, due to the limited data collection, necessitated the use of the VAS score and Barthel score for the pain level of the patients. This analysis revealed that PAD can cause severe pain and lead to a significant decrease in the ability to take care of one’s own life. The endovascular treatment resulted in a significant improvement in pain symptoms and the ability to care for oneself, compared to the preoperative period. This improvement is a notable aspect of endovascular therapy in patients with PAD.

Univariable Cox regression analysis was performed to identify factors associated with Major Adverse Limb Events (MALEs). The analysis revealed that extreme age (≥ 80 years) and complex lesion morphology (TASC II type C/D) were significantly associated with an increased risk of MALEs. This finding aligns with clinical expectations: older patients typically present with more severe vascular calcification, longer lesion lengths, and poorer distal runoff (as shown in Table 2), all of which collectively contribute to an elevated risk of restenosis and thrombosis after revascularization. Furthermore, the univariable analysis also indicated that the use of drug-coated balloons (DCB) and lipid-lowering medications served as protective factors, which is consistent with previous research findings. It is noteworthy that although factors such as stent implantation and atherectomy usage showed only borderline statistical significance (HR = 1.50, P = 0.091), their hazard ratios were higher than other factors, suggesting that a larger sample size may be needed to validate their potential impact.

## Strengths and limitations

This study has several strengths. First, it addresses a clinically significant and growing challenge in an aging population by focusing on the understudied cohort of extremely elderly patients (≥ 80 years) with CLTI. The use of propensity score matching enhances the internal validity of our findings by balancing key baseline characteristics between the extreme elderly and normal elderly groups, thereby simulating a randomized study design and reducing selection bias. Furthermore, the inclusion of patient-centered outcomes such as pain relief (VAS score) and functional capacity (Barthel Index) provides valuable insights into the qualitative benefits of endovascular therapy beyond traditional procedural success metrics.

However, several limitations must be acknowledged. The retrospective, single-center design inherently carries risks of unmeasured confounding and selection bias, despite our efforts to mitigate these through statistical adjustment. The sample size, though substantial, may still limit the statistical power to detect smaller but clinically significant differences, particularly in subgroup analyses. Importantly, due to the exploratory nature of some comparative analyses and the number of variables examined, we did not adjust P-values for multiple comparisons. Therefore, the findings from these analyses, especially those with borderline significance (e.g., P values between 0.05 and 0.10), should be interpreted as hypothesis-generating rather than confirmatory and require validation in future studies. Finally, the generalizability of our findings may be constrained by the single-center experience and specific patient population.The findings of this study must be validated in a multicenter prospective or even randomized controlled trial. Once this has been done, the identified risk factors can be further investigated and controlled in order to reduce the incidence of adverse events in extremely elderly patients.

## Conclusion

The results indicate that in cases of more severe lesions, elderly patients with PAD who undergo endovascular therapy have a higher rate of postoperative complications, adverse limb events at one year postoperatively, and all-cause mortality. However, it is important to note that endovascular therapy provides significant pain relief and improves quality of life for these patients.

## Data Availability

The original contributions presented in the study are included in the article/Supplementary Material, further inquiries can be directed to the corresponding authors.
